# The rope

**DOI:** 10.1590/2177-6709.20.6.011-012.edt

**Published:** 2015

**Authors:** David Normando

**Affiliations:** * Adjunct Professor, School of Dentistry, Universidade Federal do Pará (UFPA). Head of the graduation program in Dentistry, Universidade Federal do Pará (UFPA) and Specialization Course in Orthodontics, ABO-Pará.

Hypothesis is a tightrope swinging 

between reality and a supposed truth.

Valdeci Nogueira, Brazilian philosopher and writer.

We are aware that all information published by each issue of this journal needs the squeeze and sweat of thousands of people. On one end of the rope, researchers and higher education institutions work hard to produce science to the highest degree of excellence. On the other end, reviewers silently filter manuscripts, thus improving the quality and accuracy of information that has been approved. Science is tough, as it tests and audits one's dreams. But both ends of the rope face the same direction.

On our 19^th^ anniversary, we had to thank publicly everyone who contributed to the significant growth of this journal, especially in the last few years: researchers, reviewers and higher education institutions which believed in building a Brazilian scientific journal to excellence. The idea is not to stretch the rope, so as to create competition; but strengthen it while acknowledging all contributors' competence and thanking them for their support which is key to the development of this journal. And we did so at the 10^th^ Congress of Associação Brasileira de Ortodontia (Brazilian Association of Orthodontics- ABOR) held in Florianópolis / Santa Catarina, Brazil. At the event, we disclosed information about the last three years of the journal, reporting the participation of more than 70 higher education institutions - from Brazil, United States, Canada, Italy, England, Colombia, Syria, Turkey and Pakistan - which believed in Dental Press Journal of Orthodontics (DPJO)'s credibility. We assessed the journal's data and reported the top ten most active institutions, all of which were Brazilian: University of São Paulo (USP)/ Bauru School of Dentistry (FOB-USP), Federal University of Rio de Janeiro (UFRJ), State University of São Paulo (UNESP)/ Araraquara, State University of Maringá (UEM), State University of Southwestern Bahia (UESB), Federal University of Bahia (UFBA), Ingá College (UNINGÁ), University of São Paulo (USP)/ School of Dentistry, State University of Rio de Janeiro (UERJ), Federal University of Juiz de Fora (UFJF). All of them are acknowledged for their hard work in graduate and/or undergraduate education, and trust in our journal's earnest and quality. 

We also reported the most productive authors published in DPJO. A total of 758 researchers from dozens of countries, among which some of the most important researchers of worldwide Orthodontics contributed effusively: Guilherme Janson (FOB-USP), Antonio Carlos Ruellas (UFRJ), Karina Salvatore de Freitas (Ingá College), Ary dos Santos-Pinto (UNESP/Araraquara), Carlos Nelson Elias (Military Institute of Engineering - IME), Daniela Garib (FOB and HRAC-USP), José Fernando Castanha Henriques (FOB-USP), Rodrigo Hermont Cançado (Ingá College), Matheus Pithon (UESB), Eduardo Franzotti Sant'Anna (UFRJ), Orlando Tanaka (PUC/PR), Leopoldino Capelozza Filho (Sacred Heart University, USC) and Mauricio de Almeida Cardoso (USC). Researchers of inestimable value, pulling the rope towards DPJO and Brazilian Orthodontics growth.

If on one hand, we congratulate those who produced so much science, we certainly have to honor the other end of the rope: professionals who worked hard to filter the quality of information, so as to render it more reliable for practical applicability in society. In 2015, more than 180 reviewers, from different continents, contributed to such an indispensable task. Between 2013 and 2015, the most active were: Marcio Rodrigues de Almeida (UNOPAR), Daniela Feu Laignier (UVV-ES), Alexandre Simões da Motta (UFF), Emanuel Braga Rego (UFBA), Alex Pozzobon Pereira (UFMA), Soraya Coelho Leal (UNB), Daltro Ritter (UFSC), Andre Wilson Machado (UFBA), Hugo Caracas (Brasília/DF) and Arno Locks (UFSC). Orthodontics thanks and feels proud for all the work you have done.

If the rope has two ends, someone needs to balance the force originating in each one of them. All scientific journals have this delicate role played by the editors. At present, DPJO counts on the contribution of ten editors. They are scientists coming from different parts of Brazil as well as overseas, all dully acknowledged worldwide. We thank profusely all the work carried out by Professors Telma Martins de Araujo (UFBA), Luciane de Menezes (PUC-RS), Daniela Garib (USP), Fernanda Angelieri (UMESP) and Flavia Artese (UERJ), as well as Professors Matheus Pithon (UESB), Ildeu Andrade (PUC-MG), Leandro Silva Marques (UFVJM) and Carlos Flores-Mir (University of Alberta - Canada).

If hypothesis is a tightrope swinging between reality and a supposed truth, as described by the epigraph, we must as well be proud of our rope. Dental Press Journal of Orthodontics has operated for nearly 20 years as a strong rope supporting the bridge of scientific knowledge. The colossal extent of this bridge might bother and put us down, showing how small we are. Perhaps we are not even a rope, maybe a cord in the navel of science, only. We thus take this opportunity to realize how much we still have to grow.

David Normando - Editor-in-chief (davidnormando@hotmail.com)

